# Repetition Suppression in Ventral Visual Cortex Is Diminished as a Function of Increasing Autistic Traits

**DOI:** 10.1093/cercor/bhu149

**Published:** 2014-07-01

**Authors:** Michael P. Ewbank, Gillian Rhodes, Elisabeth A. H. von dem Hagen, Thomas E. Powell, Naomi Bright, Raliza S. Stoyanova, Simon Baron-Cohen, Andrew J. Calder

**Affiliations:** 1 Medical Research Council, Cognition and Brain Sciences Unit, Cambridge, UK; 2 ARC Centre of Excellence in Cognition and its Disorders, School of Psychology, University of Western Australia, Crawley, Western Australia, Australia; 3 Autism Research Centre, Department of Psychiatry, University of Cambridge, Cambridge, UK

**Keywords:** autism, fMRI adaptation, face-processing, fusiform, scenes

## Abstract

Repeated viewing of a stimulus causes a change in perceptual sensitivity, known as a visual aftereffect. Similarly, in neuroimaging, repetitions of the same stimulus result in a reduction in the neural response, known as repetition suppression (RS). Previous research shows that aftereffects for faces are reduced in both children with autism and in first-degree relatives. With functional magnetic resonance imaging, we found that the magnitude of RS to faces in neurotypical participants was negatively correlated with individual differences in autistic traits. We replicated this finding in a second experiment, while additional experiments showed that autistic traits also negatively predicted RS to images of scenes and simple geometric shapes. These findings suggest that a core aspect of neural function—the brain's response to repetition—is modulated by autistic traits.

## Introduction

In neuroimaging studies, repeated presentation of the same stimulus is associated with a reduction in BOLD response, known as repetition suppression (RS) or fMRI adaptation (Grill-Spector et al. 2006). RS occurs across multiple time scales, in multiple brain regions, and is found for low-level properties (e.g., color, motion) and higher level perceptual categories, such as faces (Grill-Spector et al. 2006). At a behavioral level, prolonged viewing of a particular stimulus is associated with a change in perception, commonly known as a perceptual aftereffect. Aftereffects are also found for both low- and higher level properties, including facial identity (Webster and MacLeod 2011), eye gaze (Calder et al. 2008), and facial expressions (Skinner and Benton 2010). Although it is unclear whether RS and perceptual aftereffects result from the same underlying mechanism, there is evidence to suggest that the 2 may be related (Cziraki et al. 2010; Kaiser et al. 2013).

As with all neural and psychological measures, there is substantial individual variation in the magnitude of both RS and aftereffects (Rotshtein et al. 2005; Dennett et al. 2012; Rhodes et al. 2014). However, the causes of this variation are not understood. One clue comes from research showing that face aftereffects are significantly reduced in children with autism and their first-degree relatives (Pellicano et al. 2007, 2013; Fiorentini et al. 2012). Autism is a lifelong heritable condition associated with difficulties in social communication, narrow interests, and repetitive behaviors (American Psychiatric Association 2013). One proposal is that diminished aftereffects are the consequence of less flexible perceptual coding of faces in autism (Pellicano et al. 2007). To date, no studies have examined differences in RS between people with autism and neurotypical groups in higher level category-selective regions of visual cortex. Here, we used fMRI to determine whether RS to faces is negatively related to autistic traits in neurotypical individuals and whether such a relationship extends to other object categories.

Individual differences in autistic traits have been shown to predict performance in neurotypical individuals on a number of tasks that are impaired in autism-spectrum conditions (ASC), including reading other's mental states (Baron-Cohen, Wheelwright, Hill et al. 2001), recognizing faces (Rhodes et al. 2013), local versus global processing (Grinter et al. 2009), and gaze processing (Bayliss and Tipper 2005). Since ASC is considered to be an extreme end of a normal distribution (Baron-Cohen, Wheelwright, Skinner et al. 2001; Happé et al. 2006), investigating the effect of autistic traits in neurotypical participants provides a complementary approach to studies of ASC which can be complicated by clinical comorbidity (Leyfer et al. 2006).

Given that ASC is associated with diminished face aftereffects, we predicted variation in RS to faces in neurotypical participants would be negatively correlated with individual differences in autistic traits. Autistic traits were measured with the Autism-Spectrum Quotient (AQ) (Baron-Cohen, Wheelwright, Skinner et al. 2001). To verify that the RS effects were not simply attributable to adaptation of low-level features, we measured RS across repetitions of images of the same face shown in both same and different sizes. RS to familiar (famous) and unfamiliar faces was also examined to determine whether familiarity modulated any relationship between autistic traits and RS. In a second experiment, we investigated whether the relationship between autistic traits and RS to faces could be replicated with different participants. In Experiments 3 and 4, we investigated whether a similar relationship holds for RS to nonsocial stimuli; images of scenes; and simple geometric shapes.

## Methods and Materials

### Participants

Thirty-one neurotypical volunteers participated in Experiment 1. The data from 4 participants were removed due to excessive head movement in the scanner, leaving a total of 27 participants (6 females, all right-handed, aged 18–37 years old, mean age = 24.5 years [standard deviation, SD = 6.1]). In Experiments 2 and 3, a separate set of 31 neurotypical volunteers participated. Two participants were removed due to excessive head movement, leaving a total of 29 participants (19 females, all right-handed, aged 18–39 years old, mean age = 25.5 years [SD = 6.5]). In Experiment 4, a new set of 33 typical volunteers participated. Two participants were removed due to excessive head movement, leaving a total of 31 participants (16 females, all right-handed, aged 19–39 years old, mean age = 26.6 years [SD = 5.9]). Participants were recruited through the MRC Cognition and Brain Sciences Unit's research participation system. All participants had normal or corrected-to-normal vision. None had a history of head injury, neurological, or psychiatric conditions (including autism), or was currently on medication affecting the central nervous system. The study was approved by the Cambridge Psychology Research Ethics Committee. All volunteers provided written informed written consent and were paid for participating.

### Stimuli

For all localizer scans and for Experiments 1 and 2, color photographs of unfamiliar faces with neutral expressions were obtained from the NimStim Face Stimulus Set (Tottenham et al. 2009), the Karolinska Directed Emotional Faces image set (Lundqvist and Litton 1998), and the FERET database (Phillips et al. 2000). In Experiment 1, color photographs of famous faces were obtained from the worldwide web. Familiar and unfamiliar faces were matched for gender and age, and different facial identities were used in the localizer and RS experiments. All face images were matched for interocular distance and eye position. To verify that participants could identify the famous faces, before scanning they were presented with all 84 familiar and unfamiliar faces and were asked to report their name or identifying biographical information if familiar. Mean recognition rate for familiar faces used in the RS experiment was 93.3% (SE = 2.8); mean correct rejection rate for unfamiliar faces was 97.2% (SE = 1.0). In Experiment 2, the faces were all unfamiliar and different from those used in Experiment 1. Images of scenes (Experiment 3) comprised computer-generated photorealistic interior scenes and were taken from the worldwide web. In Experiment 4, images of 8 simple geometric shapes (hexagon, triangle, rhombus, trapezoid, L-shape, circular-segment, cross, and tear) were generated using Microsoft PowerPoint 2010 and Adobe Photoshop (http://www.adobe.com).

### Localizer Scans

Participants lay supine in the magnet bore and viewed images projected onto a screen visible via an angled mirror. In Experiment 1, the localizer comprised images of 32 familiar faces, 32 unfamiliar faces, 32 houses, and 32 scrambled faces. These were presented using a block design, consisting of 4 16-s blocks for each of the 4 conditions; each block contained 8 images with each image shown for 1600 ms followed by a 400-ms blank ISI. Blocks of stimuli were separated by an 8-s rest block (fixation). The localizer scan used in Experiments 2 and 3 employed the same stimulus timings and comprised images of 64 unfamiliar faces and 64 scenes (indoor and outdoor). In Experiment 4, the localizer scan comprised images of 64 household objects, 64 scrambled versions of the objects, 64 unfamiliar faces, and 64 scenes. Each condition was presented in 8 16-s blocks separated by 8 s fixation blocks. Face-, scene-, and object-selective ROIs were identified at a minimal threshold of *P* < 0.01 uncorrected (10 contiguous voxels) using the contrasts faces > houses/scenes, scenes > faces, and objects > scrambled objects, respectively. To ensure participants were attending to all trials in the localizer scan, they performed a target detection task and responded, via a button press, whenever they saw a green dot appear on an image (15% of trials).

Prior to scanning, participants completed the AQ questionnaire, a 50-item validated measure of autistic traits that is suitable for use with neurotypical participants (Baron-Cohen, Wheelwright, Skinner et al. 2001); higher scores indicate increased numbers of autistic traits. Mean (SD) AQ scores were as follows: Experiment 1: 15.3(8.0), range: 6–36; Experiments 2 and 3: 15.2(6.7), range: 6–29; Experiment 4: 16.6(9.6) range 3–37. Across all 4 experiments, only 4 participants scored >32, a level above which 79% of individuals with high functioning autism/Asperger syndrome scored in a previous study (Baron-Cohen, Wheelwright, Skinner et al. 2001). However, the AQ is not a diagnostic measure (Baron-Cohen, Wheelwright, Skinner et al. 2001) and no participants had a clinical diagnosis of an ASC. In Experiments 1 and 4, we also performed an additional analysis excluding those participants with an AQ score >32. AQ does not correlate with measures of Intelligence Quotient in either the general or the student population (Baron-Cohen, Wheelwright, Skinner et al. 2001).

### Repetition Suppression Experiments

**Figure 1.**
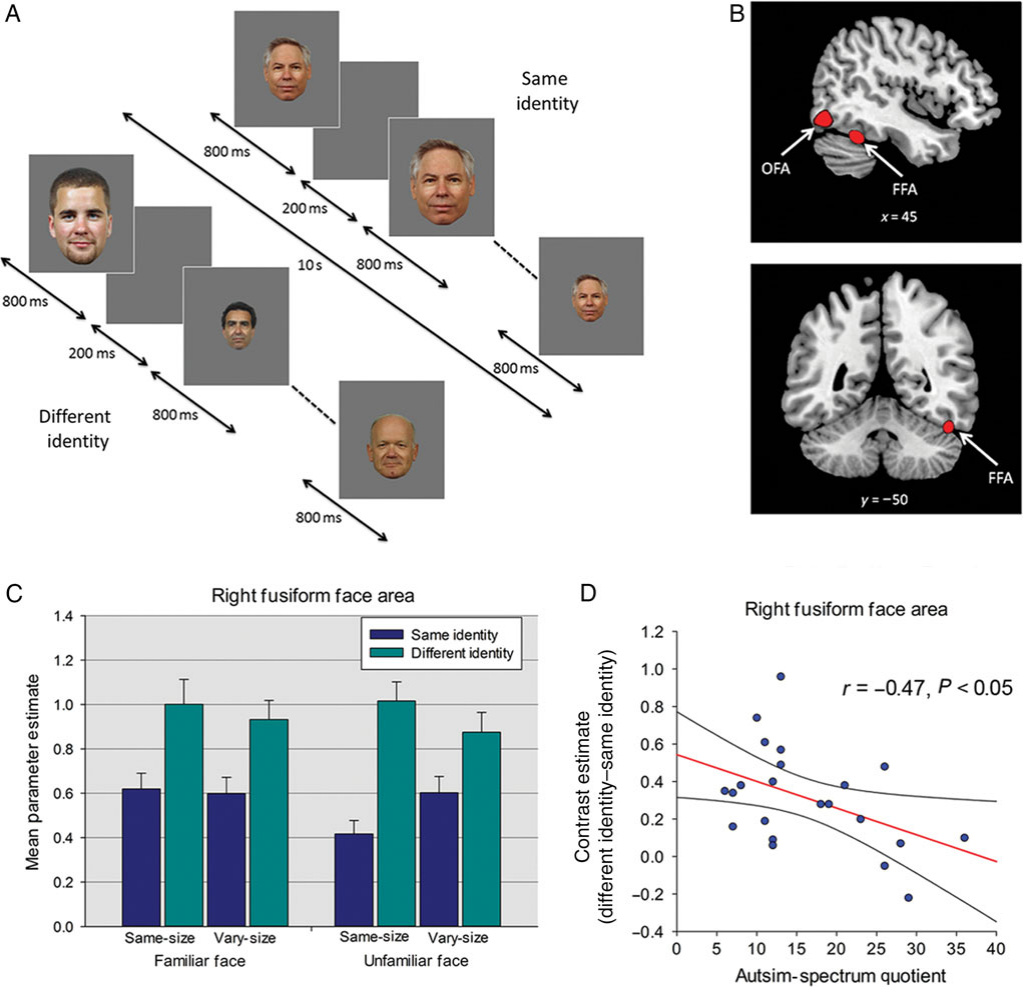
(*A*) Block-design format used in Experiment 1-RS to faces (*B*) Face-selective right FFA and right OFA from a representative participant identified with the localizer scan. (*C*) Parameter estimates (+1 SD) from Experiment 1 for each of the different- and same-identity conditions in right FFA. (*D*) Relationship between AQ and RS (different-identity–same-identity) in right FFA. Plot shows contrast estimates of RS (across Image Size and Familiarity) plotted against individual measures of AQ. The regression line and 95% confidence intervals are shown.

In Experiment 1, the RS experiment used a repeated-measures design investigating the effects of Repetition (same-identity, different-identity), Image Size (same-size, vary-size), and Familiarity (familiar faces, unfamiliar faces). Each of the 4 levels of the size and familiarity conditions (same-size familiar, vary-size familiar, same-size unfamiliar, vary-size unfamiliar) comprised 10 same-identity blocks in which the same face was shown 10 times, and 10 different-identity blocks containing images of 10 different faces; a total of 80 stimulus blocks (Fig. 1*A*). In the same-size blocks, all faces subtended a visual angle of 9° × 6°. Blocks in the vary-size condition contained images shown at full size (9° × 6°) and at 66% and 33% of this size. Each stimulus block lasted for 10 s, with each image shown for 800 ms, followed by a 200-ms blank ISI (Fig. 1*A*). Blocks of images were presented in a counterbalanced order, separated by an 8-s period of fixation when an equiluminant gray screen was shown. A total of 20 faces were used (10 familiar, 10 unfamiliar), and individual identities were shown an equal number of times in the same and different-identity blocks. Total scan time was 24 min. Participants performed a target detection task and responded, via a button press, whenever they saw a green dot appear on an image (15% of trials). In all experiments, the number of target trials was matched across same- and different-identity conditions.

Previous research suggests that reduced fusiform activity to faces in individuals with ASC relative to neurotypical controls may reflect a reduced time spent fixating the eye region of the face (Dalton et al. 2005). To determine whether autistic traits showed any relationship with dwell time on the eye region, in Experiment 1 we monitored and recorded participants' eye movements during the scanning session using a 50-Hz monocular MRI-compatible infrared eyetracker (SensoMotoric Instruments [SMI], Teltow, Germany). Eye tracking data were analyzed with SMI BeGaze3.0 software. A rectangular area of interest (AOI) was created around the eye region of the face for the full-size images and for the 66% and 33% images separately. Average dwell time in the AOI was measured for each condition (excluding trials containing targets).

**Figure 2.**
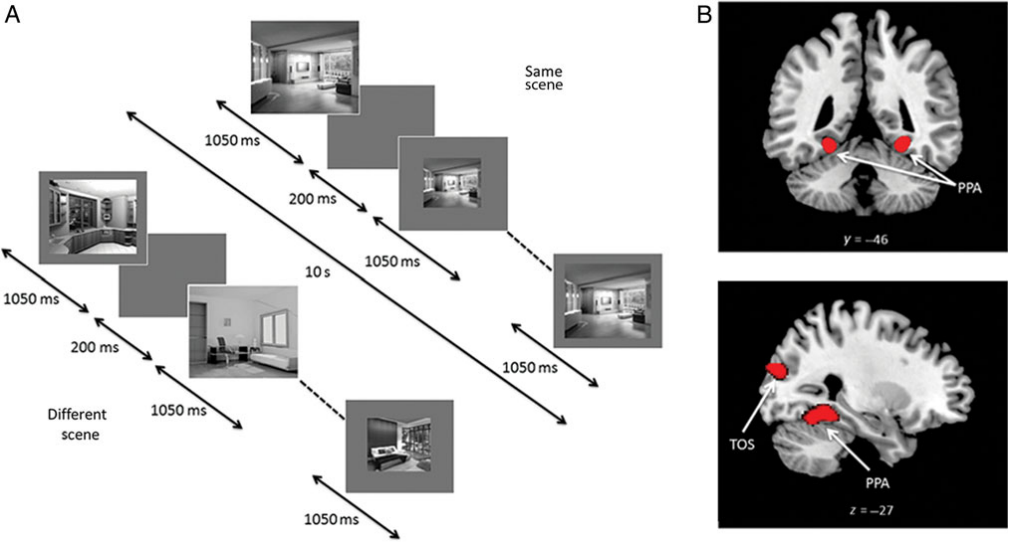
(*A*) Block-design format used in Experiments 2 and 3 (Experiment 3 shown). (*B*) Scene-selective bilateral PPA and TOS from a representative participant identified with the localizer scan.

Experiment 2 examined RS to faces and Experiment 3 examined RS to scenes. Both experiments used a repeated-measures design, investigating the effects of Repetition (same-face identity/same-scene, different-face identity/different-scene), and Image Size (same-size, vary-size). In each experiment, each of the same- and vary-size conditions contained 8 same-face identity/same-scene blocks in which the same face/scene was shown 8 times, and 8 different-face identity/different-scene blocks containing images of 8 different faces/scenes; a total of 32 stimulus blocks in each experiment. Each stimulus block lasted for 10 s, with each image shown for 1050 ms followed by a 200-ms blank ISI (Fig. 2*A*). In contrast to Experiment 1, we used longer stimulus durations in Experiments 2 and 3. This change was made in order to vary onset times relative to the TR, enabling activity to be sampled at different time points following stimulus onset, and thereby obtaining a more reliable estimate of signal change (Price et al. 1999). In both experiments, blocks of images were presented in a counterbalanced order separated by an 8-s period of fixation. To equate the amount of visual stimulation across same- and vary-size conditions, the same-size condition used face and scene images that were equivalent to the 66% sized images used in Experiment 1; Image Sizes in the vary-size condition were identical to those in Experiment 1 (i.e., 9° × 6° and 66% and 33% of this size). A total of 8 faces and 8 scenes were used and individual identities/scenes were shown an equal number of times in the same- and different-identity/scene blocks. The order of face and scene experiments was counterbalanced across participants. In both experiments, participants performed a dot-detection task (15% of images). Total scan time for each experiment was 9.6 min.

Experiment 4 used a repeated-measures design investigating the effects of Repetition (same-shape, different-shape) and Image Color (same-color, vary-color). Each of the same- and vary-color conditions contained 8 same-shape blocks in which the same shape was shown 8 times, and 8 different-shape blocks containing images of 8 different shapes; a total of 32 stimulus blocks. Timings were identical to those used in Experiments 2 and 3. Blocks of images were presented in a counterbalanced order separated by an 8-s period of fixation. Given that Experiments 1–3 found that the relationship between AQ and RS did not differ between same- and vary-size conditions, in Experiment 4 images were shown at different sizes in both the same and vary-color blocks (full-size (9° × 6°) and 66% and 33% of this size). As in the previous experiments, individual shapes were shown an equal number of times in the same- and different-shape blocks, and participants were required to perform a dot-detection task (15% of images). Total scan time was 9.6 min.

### Imaging Parameters

For Experiment 1, MRI scanning was performed on a Siemens Tim Trio 3-Tesla MR scanner with a 12-channel head coil. Brain data were acquired with *T*_2_*-weighted echo-planar imaging sensitive to BOLD signal contrast (32 slices; voxel size 3 × 3 × 1.8 mm; gap 25%; FOV 192 × 192 mm; flip angle 78°; TE 30 ms; TR 2 s). Slices were acquired sequentially in an axial orientation aligned along the ventral temporal lobes. The first 3 volumes were discarded to allow for the effects of magnetic saturation. A high-resolution structural magnetization prepared rapid gradient echo scan was also acquired at a resolution of 1 × 1 × 1 mm. Imaging parameters in Experiments 2–4 were similar, with the exception that MRI scanning was performed with a 32-channel head coil and each image volume consisted of 32, 3-mm thick slices (voxel size 3 × 3 × 3 mm).

### FMRI Analysis

Data were analyzed using SPM 8 software (Wellcome Trust Centre for Neuroimaging, London, UK). Standard preprocessing was applied, including correction for slice timing and head motion. Each participant's scans were normalized using the linear and nonlinear normalization parameters estimated from warping the participant's structural image to the Montreal Neurological Institute (MNI)—ICBM avg152 *T*_1_-weighted template, using 2-mm isotropic voxels and smoothed with a Gaussian kernel of 8 mm full-width half-maximum. Blocks of each condition were modeled by sustained epochs of neural activity (boxcars) convolved with a canonical hemodynamic response function. Realignment parameters were also included as effects of no interest to account for motion-related variance. A high pass filter of 128 s was used to remove low-frequency noise.

For Experiments 1 and 2 addressing RS to faces, mean parameter estimates for each condition were extracted from an 8-mm radius sphere centered on the maximal voxel in each participant's face-selective occipital face area (OFA) and fusiform face area (FFA) using MarsBar (Brett et al. 2002). In Experiment 3, mean parameter estimates for RS to scenes were extracted from 8-mm radius spheres centered on the maximal voxel of each participant's scene-selective transverse occipital sulcus (TOS) and parahippocampal place area (PPA). In Experiment 4, mean parameter estimates were extracted from 8-mm radius spheres centered on the maximal voxel of each participant's lateral occipital object area (LO) and posterior fusiform object area (pFs).

In Experiment 1, parameter estimates for each ROI were entered into ANCOVAs including Repetition (same-identity, different-identity), Image Size (same-size, vary-size), and Familiarity (familiar faces, unfamiliar faces) as repeated-measures factors. Experiments 2 and 3 included Repetition (same-face identity/same-scene, different-face identity/different-scene) and Image Size (same-size, vary-size) as repeated-measure factors. Experiment 4 included Repetition (same-shape, different-shape) and Image Color (same-color, different-color) as repeated-measures factors. In all studies, AQ scores and age were entered as covariates. Although we did not set out to investigate the effects of participants' sex, sex of participant was included in an initial analysis of each experiment's data. There were no effects or interactions with sex in any of the experiments; hence, this factor was dropped from the reported analyses.

Note that the analyses of the RS experiments were conducted on data extracted from ROIs that were independently defined using localizer scans. Hence, voxel selection was blind to any relationship between these voxels and AQ, avoiding the logical and statistical biases that may lead to inflated correlations (Vul et al. 2009; Calder et al. 2011).

As the absolute difference between same- and different-identity conditions could potentially increase as a function of the overall response, we established whether the relationship between AQ and RS was accounted for by a more fundamental relationship between AQ and the overall response to faces, scenes, or simple shapes by calculating an Adaptation Index (AI) for each participant in each experiment. For the AI, RS was measured as the difference between different- and same-identity conditions relative to the summed response in both conditions:

$$\begin{array}{ll} & \text{AI = }\frac{\text{Different Identity}-\mathrm{Same}\;\mathrm{Identity}}{|\text{Different Identity + Same Identity}|} \end{array}$$

Any relationship between AQ and RS effects could also potentially be a consequence of differences in category selectivity in a region (e.g., difference between faces and houses in FFA). To determine whether this was the case, we performed a correlation analysis examining the relationship between individual AQ scores and category selectivity in face-, scene-, and object-selective regions.

As well as incorporating movement parameters into our first-level analysis, to determine whether any relationship between AQ and RS could be explained by differences in participant movement during the scan, we also calculated root-mean-square movement across all translation and rotation directions for each participant and entered these data into a correlation analysis. This revealed no significant relationship between AQ and mean movement in any of the 4 experiments (Exp 1: [*r* = 0.18, *P* = 0.38]; Exp 2: [*r* = 0.20, *P* = 0.28]; Exp 3: [*r* = 0.14, *P* = 0.47]; Exp 4: [*r* = −0.04, *P* = 0.98]). Hence, any relationship between AQ and RS in the results is unlikely to be driven by differences in participant movement during the scan.

Finally, in each experiment, we determined whether RS occurred outside face-, scene-, or object-selective regions by performing a whole-brain analysis (different-identity > same-identity) (*P* < 0.05 FWE corrected, 10 contiguous voxels) across all participants. Similarly, to determine whether regions outside of the category-selective ROIs showed a relationship between RS and AQ, single-participant-level contrasts (different-identity > same-identity) were entered into a whole-brain, group-level regression analysis with AQ and age as covariates.

## Results

### Experiment 1: Localizer Scan

Using the contrast faces > houses, in the right hemisphere, we localized FFA in 26 of 27 participants, and OFA in 24 participants (Fig. 1*B*). In the left hemisphere, FFA was identified in 23 participants and OFA in 19 participants. Mean (SD) MNI coordinates for each ROI: right FFA: 42(3.4), −48(6.0), −20(4.2); left FFA: −41(3.0), −49(5.5), −20(3.1); right OFA: 44(5.0), −75(8.0), −11(6.7); left OFA: −42(5.1), −74(7.8), −12(4.8).

### Experiment 1: Repetition Suppression to Faces

#### ROI Analysis

Data extracted from each ROI were submitted to separate ANOVAs examining the effects of Repetition (same-identity, different-identity), Image Size (same-size, vary-size), and Familiarity (familiar faces, unfamiliar faces) as repeated-measures factors, with participants' sex as a between-subjects factor. These revealed no main effect or interactions involving sex in any region (*P*'s > 0.21). Similarly, ANOVAs of data from Experiments 2–4 including sex also showed no effects or interactions with this variable (*P*'s > 0.19). Therefore, sex was not included as a factor in any of the reported analyses.

To investigate the effect of AQ on RS, data from the 4 ROIs were submitted to separate ANCOVAs examining the effects of Repetition (same-identity, different-identity), Image Size (same-size, vary-size), and Familiarity (familiar faces, unfamiliar faces) as repeated-measures factors, and participants' AQ scores and age as covariates. For the right FFA, the ANCOVA revealed a significant effect of Repetition (RS), reflecting a greater response in the different-identity compared with the same-identity condition (*F*_1,23_ = 54.28, *P* < 0.001, *η*_ρ_^2^ = 0.70) (Fig. 1*C*). In addition, there was a significant interaction between Repetition and Image Size, reflecting greater RS in the same-size condition than the vary-size condition (*F*_1,23_ = 9.71, *P* < .005, *η*_ρ_^2^ = 0.30). This was qualified by a significant 3-way interaction between Repetition, Image Size, and Familiarity, reflecting greater generalization of RS across size for familiar faces relative to unfamiliar faces (*F*_1,23_ = 7.65, *P* < 0.05, *η*_ρ_^2^ = 0.25). Importantly, there was a significant interaction between AQ and Repetition, reflecting diminished RS as a function of increasing AQ scores (*F*_1,23_ = 6.90, *P* < 0.05, *η*_ρ_^2^ = 0.23) (Fig. 1*D*). This interaction was not further modulated by 3-way or 4-way interactions with Image Size and/or Familiarity (*P*'s > 0.42) and there were no effects or interactions involving age (*P*'s > 0.23) (note that the interaction between AQ and Repetition remained significant when age was excluded from the ANCOVA; the same applies to all reported interactions between AQ and Repetition in subsequent experiments). Similar ANCOVAs for each additional ROI showed significant effects of Repetition in the left FFA and right and left OFA (*P*'s < 0.05). However, RS in these regions did not interact with AQ (*P*'s > 0.25) and there was no interaction involving age in any of these regions (*P*'s > 0.12).

One possibility is that the relationship between AQ and Repetition reflects a more fundamental relationship between AQ and the overall neural response to faces; this is because the absolute difference between same and different-identity conditions could increase as a function of the overall response to faces (i.e., a scaling effect). To investigate this possibility, we calculated an AI (i.e., RS as a proportion of the overall response in both same- and different-identity conditions, see Materials and Methods) for each participant. AI scores for right FFA were entered into an ANCOVA examining the effects of Image Size (same-size, vary-size) and Familiarity (familiar faces, unfamiliar faces) as repeated-measures factors, with AQ and age as covariates. This revealed a significant effect of AQ (*F*_1,23_ = 5.61, *P* < 0.05, *η*_ρ_^2^ = 0.20) reflecting diminished RS as a function of increasing AQ scores. This relationship was not further modulated by significant 2-way or 3-way interactions with Image Size and/or Familiarity (*P*'s > 0.10). There was no main effect or interactions involving age (*P*'s > 0.55) and no effect of AQ in other face-selective ROIs (*P*'s > 0.38).

Another possible explanation for these findings is that the relationship between AQ and RS to faces in right FFA may reflect a relationship between AQ and face selectivity in this region. However, a correlation analysis revealed no significant relationship between AQ and face selectivity (Faces > Houses) in right FFA (*r* = −0.21, *P* = 0.29). Thus, individual differences in selectivity and the overall response to faces are unlikely to account for the relationship between AQ and RS in this region.

Although autistic traits are considered a continuum that extends from the neurotypical population into the population of people with autism, in a previous study 79% of individuals with high functioning autism/Asperger syndrome scored >32 on the AQ (Baron-Cohen, Wheelwright, Skinner et al. 2001). To determine whether the negative relationship between AQ and RS was driven by participants scoring in this range, we performed the analysis excluding individuals with an AQ score above 32. Again, this revealed a significant interaction between AQ and RS in right FFA for the raw values (*F*_1,22_ = 5.79, *P* < 0.05, *η*_ρ_^2^ = 0.21) and a main effect of AQ for the AI (*F*_1,22_ = 5.26, *P* < 0.05, *η*_ρ_^2^ = 0.19).

#### Whole-Brain Analysis

A whole-brain analysis revealed that outside of OFA and FFA, no other regions showed RS (i.e., greater response to different-identity compared with same-identity) that survived correction for multiple comparisons (*P* < 0.05 FWE). To explore whether regions other than the FFA showed a relationship between AQ and RS, we performed a whole-brain regression analysis (*P* < 0.05 FWE), including AQ and age as covariates. This revealed a negative relationship between AQ and RS in 2 areas, one corresponding to the right inferior frontal gyrus (34, 30, −10, *t*_(23)_ = 6.28, *P* < 0.05 FWE) and another centered on the dorsal surface of the cerebellum and extending into the ventral precuneus (2, −52, 6, *t*_(23)_ = 6.57, *P* < 0.05 FWE). There was no evidence of a positive relationship between AQ and RS.

#### Behavioral Data and Attentional Focus

Accuracy rates for the dot-detection task were high (mean [SD] = 99.1% [2.9]) and were not analyzed further. Mean RT (SD) across all conditions was 451.3 ms (42.7). A correlation analysis revealed no relationship between AQ and RTs to detect targets in either the same-identity or different-identity conditions (*P*'s > 0.80), suggesting that AQ was not related to participants' attention to the faces.

In light of previous work showing that the response to faces in the fusiform gyrus of individuals with autism is related to the amount of time spent attending the eyes (Dalton et al. 2005), we also examined whether there was a relationship between AQ and dwell time on the eye region of the face. Due to difficulties in tracking some participants' pupils (e.g., drooping eyelids, corrective lenses), reliable eye tracking data were only available from 12 participants. A correlation analysis revealed no significant relationship between AQ and dwell time on the eye region of the face in these participants (*P* = 0.54). Consistent with the main analysis, an ANCOVA on this subset of participants examining Repetition, Image Size, and Familiarity as repeated-measures factors, with AQ, age, and dwell time included as covariates, revealed a significant interaction between AQ and Repetition in right FFA (*F*_1,11_ = 4.61, *P* = 0.05, *η*_ρ_^2^ = 0.30). Again, the relationship between AQ and Repetition was not modulated by significant 3-way or 4-way interactions with Familiarity and/or Image size, and there were no interactions involving age (*P*'s > 0.32). Importantly, there was no significant interaction between dwell time and Repetition (*P* = 0.53). Together with the behavioral data, the results suggest that differences in attention or gaze fixations are unlikely to explain the negative relationship between AQ and RS in the right FFA.

### Experiments 2 and 3

Experiment 2 investigated whether the negative relationship between AQ and RS to faces could be replicated in a different group of participants. Since there was no effect of face familiarity on the AQ–RS relationship in Experiment 1, only unfamiliar faces were used. Experiment 3, with the same participants, determined whether the relationship between AQ and RS extended to another visual category, images of indoor scenes (Fig. 2*A*).

### Experiments 2 and 3: Localizer Scan

Using the contrast faces > scenes, we localized FFA in the right hemisphere in 27 of 29 participants and right OFA in all 29 participants. In the left hemisphere, FFA was identified in 27 participants and OFA in 21 participants. Using the contrast scenes > faces, PPA and TOS were localized bilaterally in all 29 participants (Fig. 2*B*). Mean (SD) MNI coordinates for each ROI: right FFA: 41(3.0), −48(7.1), −19(4.5); left FFA: −41(2.6), −46(6.2), −22(3.4); right OFA: 43(3.8), −73(8.3), −12(5.4); left OFA: −40(4.1), −72(8.4), −13(7.7); right PPA: 29(2.6), −46(5.5), −9(2.5); left PPA: −27(2.9), −48(5.4), −10(3.3); right TOS: 36(3.8), −83(3.5), 16(6.2); left TOS: −33(3.4), −87(3.6), 15(6.4).

### Experiment 2: Repetition Suppression to Faces

#### ROI Analysis

**Figure 3.**
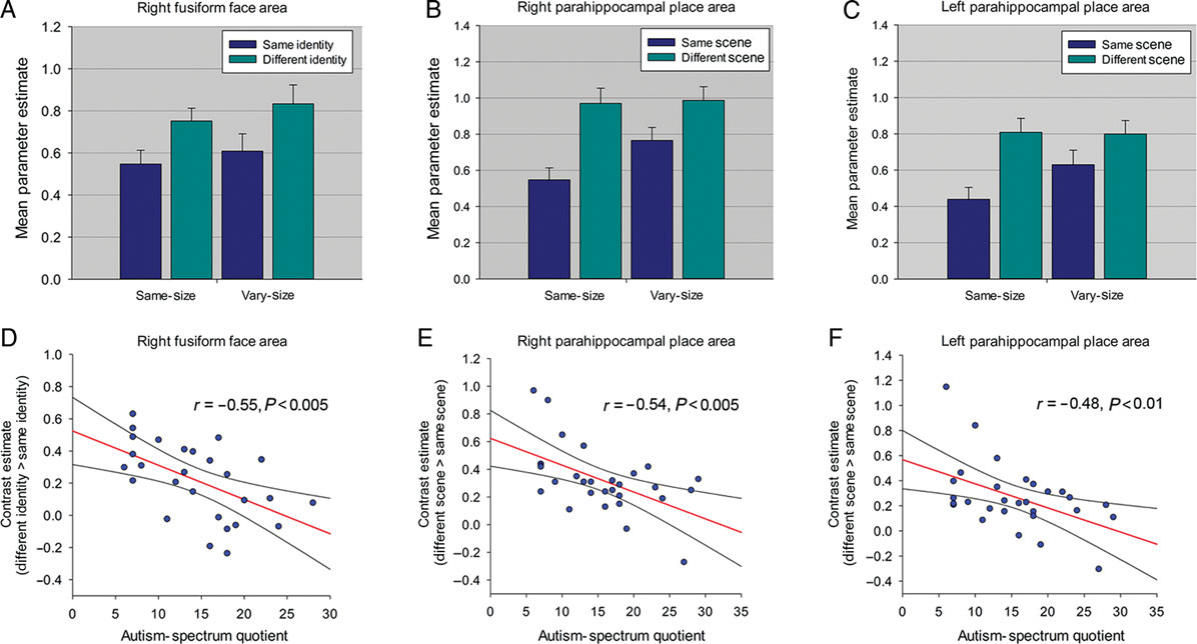
(*A*) Parameter estimates (+1 SD) for same- and different-identity conditions in right FFA in Experiment 2. (*B*) Parameter estimates (+1 SD) for all same- and different-scene conditions in right PPA and (*C*) left PPA in Experiment 3. (*D*) Relationship between AQ and RS in right FFA in Experiment 2. (*E*) Relationship between AQ and RS in right PPA and (*F*) left PPA in Experiment 3. All scatter plots show contrast estimates of RS (across Image Size) plotted against individual measures of AQ. Regression line and 95% confidence intervals are shown.

For each ROI, ANCOVA examined the effects of Repetition (same-identity, different-identity) and Image Size (same-size, vary-size) as repeated-measures factors, with participants' AQ scores and age as covariates. For the right FFA, this revealed a significant effect of Repetition, reflecting a greater response in the different-identity condition compared with the same-identity condition (*F*_1,24_ = 25.09, *P* < 0.001, *η*_ρ_^2^ = 0.51) (Fig. 3*A*), but no interaction between Repetition and Image Size (*P* = 0.88). Consistent with Experiment 1, there was a significant interaction between AQ and Repetition, reflecting reduced RS with increasing AQ scores (*F*_1,24_ = 9.65, *P* < 0.005, *η*_ρ_^2^ = 0.29) (Fig. 3*D*). This interaction was not further modulated by a 3-way interaction with Image Size (*P* = 0.30) and there were no interactions involving age (*P*'s > 0.80).

Similar ANCOVAs for each additional face-selective ROI revealed a main effect of Repetition in left FFA and bilateral OFA (all *P*'s < 0.001). There was also an interaction between AQ and Repetition in left OFA (*F*_1,18_ = 6.13, *P* < 0.05, *η*_ρ_^2^ = 0.25) and a borderline interaction in right OFA (*F*_1,26_ = 3.11, *P* = 0.06, *η*_ρ_^2^ = 0.13), reflecting negative relationships between AQ and Repetition; neither was qualified by a significant 3-way interaction with Image Size (*P*'s > 0.43). In addition, there were no interactions involving age (*P*'s > 0.25). There was no interaction between AQ and Repetition in left FFA (*P* = 0.31).

As in Experiment 1, we calculated an AI (i.e., RS as a proportion of the overall response in both same- and different-identity conditions). Using the AI, an ANCOVA with Image Size as a repeated-measures factor, and participants' AQ scores and age as covariates, revealed a significant effect of AQ in right FFA (*F*_1,24_ = 4.35, *P* < 0.05, *η*_ρ_^2^ = 0.15), reflecting diminished RS as a function of increasing AQ; this was not further modulated by a significant 2-way interaction with Image Size (*P* = 0.39). There was also no significant effect or interactions involving age (*P*'s > 0.37). Similar ANCOVAs revealed no significant effect of AQ in right and left OFAs (*P*'s > 0.13), indicating that the relationship between AQ and RS in the main analysis was no longer significant when using the AI as a measure of RS. There was also no effect of AQ in left FFA (*P* = 0.62). Finally, a correlation analysis revealed no significant relationship between AQ and face selectivity in right FFA (*r* = −0.30, *P* = 0.12). Hence, as in Experiment 1, the results suggest that the negative relationship between AQ and RS to faces is most evident in the right FFA, and that the relationship in this region is unlikely to be accounted for by variation in face selectivity or the overall response to faces in this region.

#### Whole-Brain Analysis

A whole-brain analysis revealed that no regions outside the OFA and FFA showed RS that survived correction for multiple comparisons (*P* < 0.05 FWE). A whole-brain regression analysis, including AQ and age as covariates, revealed no further regions showing either a negative or positive relationship between AQ and RS.

#### Behavioral data

As in Experiment 1, accuracy rates for the dot-detection task were high, so were not analyzed further because of ceiling effects (mean [SD] = 98.2% [4.9]. Mean RT (SD) across all conditions was 466.2 (58.6) ms. A correlation analysis revealed no evidence of a relationship between AQ and RTs to detect targets in the same- or different-identity conditions (*P*'s > 0.49), again suggesting that AQ was not related to participants' attention to faces.

### Experiment 3: Repetition Suppression to Scenes

#### ROI Analysis

ANCOVAs examined the effects of Repetition (same-scene, different-scene) and Image Size (same-size, vary-size) as repeated-measures factors, with participants' AQ scores and age as covariates. This revealed a significant effect of Repetition in all regions (right PPA [*F*_1,26_ = 74.79, *P* < 0.001, *η*_ρ_^2^ = 0.74]; left PPA [*F*_1,26_ = 40.29, *P* < 0.001, *η*_ρ_^2^ = 0.61]; right TOS [*F*_1,23_ = 17.49, *P* < 0.001, *η*_ρ_^2^ = 0.40]; left TOS [*F*_1,26_ = 17.19, *P* < 0.001, *η*_ρ_^2^ = 0.40]) reflecting a greater response in the different-scene compared with the same-scene condition. There was also an interaction between Repetition and Image Size in all regions (right PPA [*F*_1,26_ = 8.36, *P* < 0.01, *η*_ρ_^2^ = 0.23]; left PPA [*F*_1,26_ = 7.87, *P* < 0.01, *η*_ρ_^2^ = 0.23]; right TOS [*F*_1,26_ = 3.88, *P* = 0.05, *η*_ρ_^2^ = 0.13]; left TOS [*F*_1,26_ = 5.25, *P* < 0.05, *η*_ρ_^2^ = 0.17]) reflecting greater RS in the same-size condition relative to the vary-size condition (Fig. 3*B* and *C*). Importantly, there was a significant interaction between AQ and Repetition in all regions, reflecting reduced RS with increasing AQ scores in right PPA (*F*_1,26_ = 12.45, *P* < 0.005, *η*_ρ_^2^ = 0.33) and left PPA (*F*_1,26_ = 9.87, *P* < 0.005, *η*_ρ_^2^ = 0.28) (Fig. 3*E* and *F*), and in right TOS (*F*_1,26_ = 9.84, *P* < 0.005, *η*_ρ_^2^ = 0.28) and left TOS (*F*_1,26_ = 8.43, *P* < 0.01, *η*_ρ_^2^ = 0.25). The interaction between AQ and Repetition in each ROI was not further modulated by significant 3-way interactions with Image Size (*P*'s > 0.36), and there were no significant interactions involving age (*P*'s > 0.10).

ANCOVAs using the AI as a measure of RS, revealed a significant effect of AQ in all regions (right PPA [*F*_1,26_ = 6.81, *P* < 0.05, *η*_ρ_^2^ = 0.21]; left PPA [*F*_1,26_ = 7.57, *P* < 0.05, *η*_ρ_^2^ = 0.23]; right TOS [*F*_1,26_ = 7.72, *P* < 0.05, *η*_ρ_^2^ = 0.23]; left TOS [*F*_1,26_ = 6.62, *P* < 0.05, *η*_ρ_^2^ = 0.20]). These effects were not further modulated by a significant 2-way interaction with Image Size (*P*'s > 0.54) and there were no significant effects or interactions involving age (*P*'s > 0.08). Finally, a correlation analysis revealed no significant relationship between AQ and scene selectivity (Scenes > Faces) in right PPA (*P* = 0.37) or left or right TOS (*P*'s > 0.10). There was a significant relationship between AQ and scene selectivity in left PPA (*r* = −0.37, *P* < 0.05). However, given that a relationship between AQ and RS was found in both left and right PPA and left and right TOS, these findings suggest differences in selectivity or the overall response are unlikely to account for the relationship between AQ and RS to scenes.

#### Whole-Brain Analysis

A whole-brain analysis revealed that outside of scene-selective regions, RS was also found in bilateral precuneus (RH: 16, −54, 14, *t*_(28)_ = 6.28, *P* < 0.05 FWE; LH: −20, −60, 18, *t*_(28)_ = 6.42, *P* < 0.05 FWE)*.* A regression analysis including AQ and age as covariates revealed that no further regions showed either a negative or positive relationship between RS and AQ that survived correction for multiple comparisons.

#### Behavioral data

Accuracy rates for the dot-detection task were high and were therefore not analyzed further (mean [SD] = 97.2% [5.9]). Mean RT (SD) across all condition was 516.7 (73.2) ms. A correlation analysis revealed no evidence of a relationship between AQ and RTs to detect targets in the same- or different-identity conditions (*P*'s > 0.44), suggesting that AQ was not related to participants' attention to scenes.

### Experiment 4

Compared with other object categories, face perception depends more on holistic or configural processing than part-based processing (Young et al. 1987; McKone et al. 2007; Rhodes 2012). Like faces, scenes are also composed of multiple components and the overall meaning or “gist” of a scene is generally extracted before the component parts (Biederman 1981). Given that ASC has been associated with a bias toward local or feature-based processing (as opposed to global/holistic processing) (Happé and Frith 2006), one possibility is that diminished RS to faces or scenes in participants with a high number of autistic traits might reflect a greater reliance on local features. This reliance could result in attention to different local features in successive presentations of the same stimulus, leading to reduced RS. To address this possibility, in Experiment 4 we measured RS to simple geometric shapes that each comprised a single contour and minimal local features. Evidence of a negative relationship between AQ and RS to simple shapes would suggest that the relationship between autistic traits and RS is less likely to reflect a local bias.

**Figure 4.**
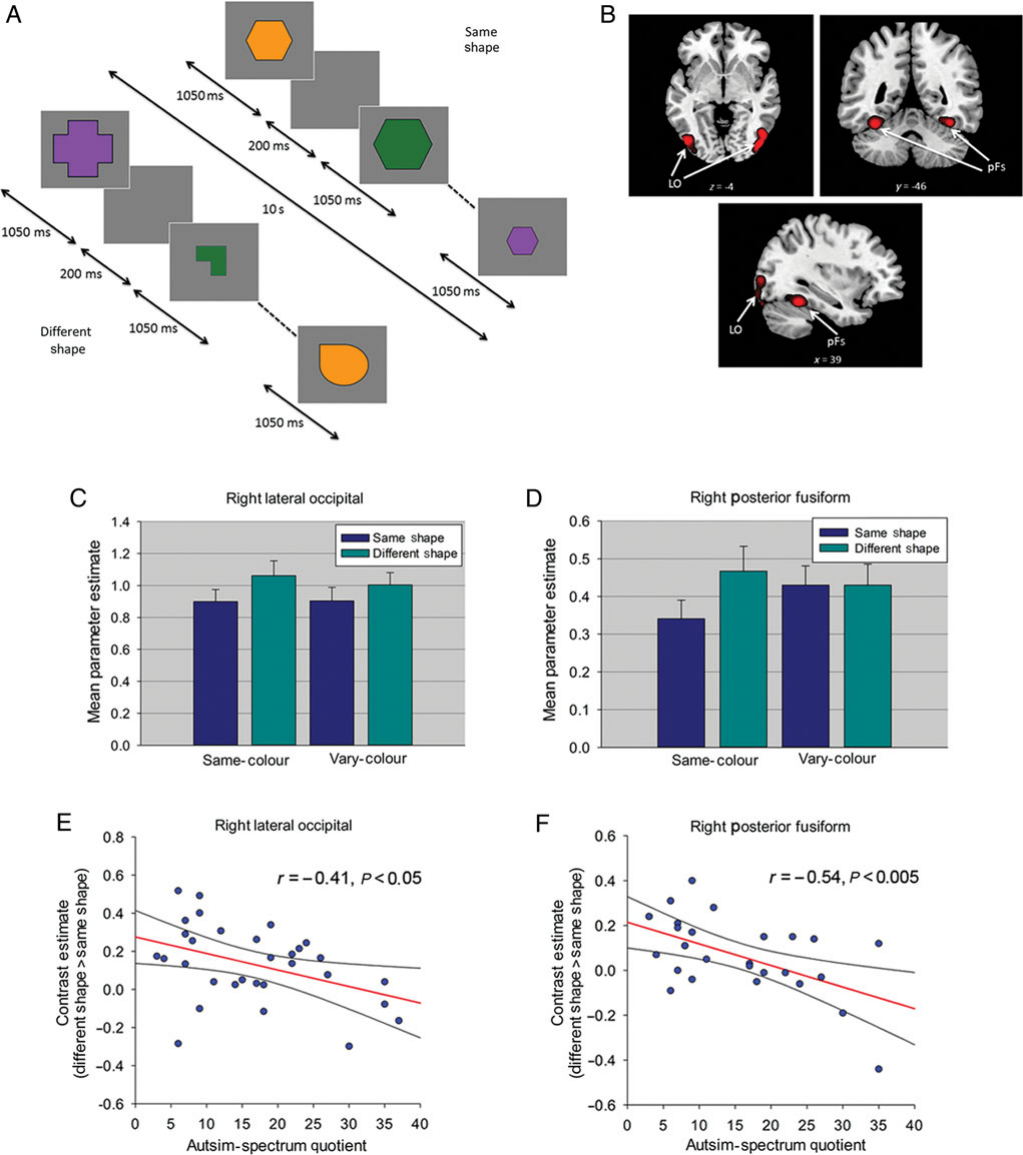
(*A*) Block-design format used in Experiment 4. (*B*) Object-selective LO and pFs from a representative participant identified with the localizer scan. (*C*) Parameter estimates (+1 SD) for same- and different-shape conditions in right LO and (*D*) right pFs in Experiment 4. (*E*) Relationship between AQ and RS in right LO and (*F*) right pFs in Experiment 4. Both scatter plots show contrast estimates of RS (across Color) plotted against individual measures of AQ. Regression line and 95% confidence intervals are shown.

As Experiments 1–3 revealed no effect of Image Size on the relationship between RS and AQ, in Experiment 4 we varied Image Size in all blocks thereby minimizing any contribution from low-level adaptation. Additionally, if reduced RS to faces and scenes in high autistic trait participants is due to them attending to different aspects of the stimulus on successive presentations, then changing a feature or attribute of a repeated shape across trials (i.e., color) should lead to a greater reduction in RS in high autistic trait participants than when the feature or attribute is kept constant. To test this possibility, we included blocks in which repeated (and different) shapes were shown in the same color or in different colors (Fig. 4*A*). We focused on 2 object-selective regions of occipitotemporal cortex; the LO and the pFs.

### Experiment 4: Localizer Scan

Using the contrast objects > scrambled objects, in the right hemisphere we localized LO in 30 of 31 participants and pFs in 27 participants. In the left hemisphere, LO was identified in 29 and pFs in 27 participants (Fig. 4*B*). Mean (SD) MNI coordinates for each ROI: right LO: 42(3.7), −76(7.0), −9(4.2); left LO: −43(4.3), −76(7.9), −7(4.7); right pFs: 36(2.9), −45(4.6), −20(3.0); left pFs: −35(4.5), −47(5.9), −18(3.9).

### Experiment 4: Repetition Suppression to Simple Geometric Shapes

#### ROI Analysis

ANCOVAs examined the effects of Repetition (same-shape, different-shape) and Image Color (same-color, vary-color) as repeated measure factors, with participants' AQ scores and age as covariates. This revealed a significant effect of Repetition (RS) in right LO (*F*_1,27_ = 14.42, *P* < 0.005, *η*_ρ_^2^ = .35), left LO (*F*_1,26_ = 18.65, *P* < 0.001, *η*_ρ_^2^ = 0.42), left pFs (*F*_1,24_ = 8.06, *P* < 0.01, *η*_ρ_^2^ = 0.25), and a borderline effect in right pFs (*F*_1,24_ = 3.11, *P* = 0.09, *η*_ρ_^2^ = 0.12) (Fig. 4*C* and D). There was no interaction between RS and Image Color in any ROI (*P*'s > 0.21). ANCOVAs also revealed an interaction between AQ and Repetition in right LO (*F*_1,27_ = 6.68, *P* < 0.05, *η*_ρ_^2^ = 0.20) and right pFs (*F*_1,24_ = 10.37, *P* < 0.01, *η*_ρ_^2^ = 0.30), again reflecting diminished RS as a function of increasing AQ scores (Fig. 4*E* and *F*). These interactions were not further modulated by 3-way interactions with Image Color, and there were no interactions involving age in either region (*P*'s > 0.22). There was no significant interaction between AQ and Repetition in either left LO or left pFs (*P*'s > 0.16).

ANCOVAs including the AI as a measure of RS revealed a significant effect of AQ in right LO (*F*_1,27_ = 7.58, *P* < 0.05, *η*_ρ_^2^ = 0.22) and right pFs (*F*_1,24_ = 4.12, *P* = 0.05, *η*_ρ_^2^ = 0.15). There was also a significant effect of AQ in left LO (*F*_1,26_ = 4.27, *P* < 0.05, *η*_ρ_^2^ = 0.14), but not left pFS (*P* = 0.13). This effect was not modulated by an interaction with Image Color in any ROI (*P*'s > 0.24). After excluding participants with AQ scores above 32, the interaction between AQ and RS remained significant in right pFs: raw values (*F*_1,21_ = 5.56, *P* < 0.05, *η*_ρ_^2^ = 0.21); AI (*F*_1,21_ = 5.43, *P* < 0.05, *η*_ρ_^2^ = 0.21), and in right LO: raw values (*F*_1,24_ = 4.01, *P* = 0.056, *η*_ρ_^2^ = 0.14); AI (*F*_1,24_ = 5.46, *P* < 0.05, *η*_ρ_^2^ = 0.19).

Finally, a correlation analysis revealed no significant relationship between AQ and object selectivity (objects vs. scrambled objects) in bilateral pFs (*P*'s > 0.56) or right LO (*P* = 0.09), with a borderline relationship in left LO (*P* = 0.06). Thus, differences in category selectivity or the overall response to shapes in high AQ participants are unable to explain the significant relationship between AQ and RS found in right LO and right pFs.

#### Whole-Brain Analysis

A whole-brain analysis revealed that outside of LO and pFs, no other regions showed RS that survived correction for multiple comparisons (*P* < 0.05 FWE). A regression analysis including AQ and age as covariates revealed no further regions showing either a negative or positive relationship between RS and AQ that survived correction for multiple comparisons.

#### Behavioral data

As in the previous experiments, accuracy rates for the dot-detection task were high (mean [SD] = 98.9% [0.3]) and were not analyzed further. Mean RT (SD) across all conditions was 478.5 (73.1) ms. A correlation analysis revealed no evidence of a significant relationship between AQ and RTs in the same- or different-shape conditions (*P*'s > 0.36) providing no evidence that AQ scores were related to participants' attention to simple geometric shapes.

Experiment 4 showed that RS to simple geometric shapes was negatively related to autistic traits. Taken together with the findings of Experiments 1–3, this suggests that the relationship between autistic traits and RS is not restricted to faces, but extends to both complex and simple nonsocial stimuli (i.e., scenes and simple shapes). In addition, the relationship between autistic traits and RS to shapes did not statistically differ when the color of the shapes in any given block remained constant or varied. Hence, it seems unlikely that reduced RS to faces, scenes, or shapes in high autistic trait participants is due to them attending to different object features, or focusing on aspects of the stimuli that are different, on successive trials in each block.

## Discussion

Variation in autistic traits has been proposed to constitute a continuum that extends from individuals with ASC into the neurotypical population (Baron-Cohen, Wheelwright, Skinner et al. 2001). Our results show that the magnitude of RS in neurotypical participants is related to individual differences in autistic traits. Experiment 1 showed that RS to faces in FFA was diminished, or effectively abolished, as a function of increasing autistic traits. We replicated these findings in different participants in Experiment 2 and also found that autistic traits were negatively correlated with RS to scenes in bilateral PPA and TOS (Experiment 3). In a third set of participants, we found a negative relationship between autistic traits and RS to simple geometric shapes in object-selective LO and posterior fusiform gyrus (Experiment 4). In all experiments, the relationship between RS and autistic traits was present across changes in Image Size, suggesting that these effects are unlikely to reflect differences in adaptation of low-level visual properties. Taken together, the findings suggest that reduced RS in higher level category-selective cortical areas may be a neural signature of increased autistic traits.

Consistent with the dominant role of the right hemisphere in face perception (Rhodes 1985), the relationship between autistic traits and RS to faces in Experiments 1 and 2 was found in the right but not left FFA. Previous research has shown that ASC is associated with reduced activation to faces in FFA, although this pattern is not consistently found (see Golarai et al. (2006) for a review). In addition, Dalton et al. (2005) have shown that a reduced fusiform response to faces in ASC could reflect reduced fixations on the eye region rather than reduced involvement of this region in processing faces. The experiments examining RS to faces, however, showed that the relationship between autistic traits and RS in FFA could not be accounted for by variation in the overall response to faces, as shown by the analysis of the AI, or by differences in face selectivity. Furthermore, Experiment 1 found that the significant relationship between autistic traits and RS to faces was unlikely to be accounted for by the amount of time spent looking at the eyes in participants with eye tracking data. Finally, the absence of an effect of face familiarity in Experiment 1 suggests that the difference in RS between high and low autistic trait participants occurs at the level of perceptual encoding of faces, rather than established representations of individual facial identities; however, additional experiments are required to confirm this interpretation.

In behavioral studies, ASC has been associated with difficulties in face recognition and memory, although the extent to which these impairments are specific to faces is unclear (Weigelt et al. 2012; Ewing et al. 2013b). Here, the relationship between autistic traits and RS was not selective to faces, as similar patterns were also found for nonsocial scenes and simple geometric shapes, which are not typically associated with difficulties in individuals with ASC. Indeed, the neural response to scenes in PPA appears to be similar in ASC and neurotypical participants (Bird et al. 2006; Humphreys et al. 2008; Kleinhans et al. 2008; Scherf et al. 2010), although it should be noted that relatively few studies have investigated the response to scenes/buildings in ASC. Similarly, we are not aware of any data showing an atypical neural response to simple geometric shapes in people with ASC. Consistent with these observations, the relationship between autistic traits and RS was also evident when using the AI as a measure of RS to scenes and shapes, suggesting that differences in the overall response to these categories do not underlie the relationship between autistic traits and RS in scene- or shape-selective regions.

Across the 4 experiments, our results suggest that autistic traits are associated with differences in the adaptive properties of higher level visual cortex coding faces, scenes, and shapes, rather than in the overall response of these regions to their preferred categories. Future work should investigate the extent to which these effects are restricted to preferred categories in each ROI; however, the current study was not optimized to address this issue, given that our ROIs were not defined exclusively for each category (i.e., FFA may contain object-selective voxels), and that data were subjected to normalization and spatial smoothing procedures before being extracted from relatively large 8-mm sphere ROIs. It will also be interesting to determine whether this relationship reflects a more general relationship between autistic traits and the adaptive properties of all brain regions. A key question is whether reduced RS constitutes a possible biomarker of autism, or a particular class of autistic symptoms. For example, might rigid and repetitive behaviors result from a failure of the brain to adapt or habituate to a particular repeated action or item of interest? An advantage of reduced RS as a potential biomarker for ASC, or a subset of symptoms associated with ASC, is that it does not rely on using the sorts of social stimuli that individuals with ASC typically have difficulty with.

Investigating the influence of autistic traits in neurotypical participants provides a complementary approach to studies of individuals with ASC, and allows for a quantitative approach rather than one based upon categorical diagnosis (Baron-Cohen, Wheelwright, Skinner et al. 2001). A key next stage, however, is to determine whether RS is also markedly reduced in individuals with a clinical diagnosis of ASC. Another study is underway in our lab testing RS in individuals with a formal diagnosis of ASC, who we predict should show markedly reduced or abolished RS. In line with this, recent work from Jiang et al. (2013) suggests that individuals with ASC show decreased release from adaptation in FFA for small face shape differences relative to that shown by neurotypical participants in an earlier study (Jiang et al. 2006) (although it should be noted they did not directly compare the 2 groups). However, Dinstein et al. (2010) did not find reduced RS in ASC in primary cortices when participants were viewing or performing hand movements, suggesting diminished RS may be restricted to higher level visual areas.

Previous work has found that individuals with ASC show reduced habituation to faces in bilateral amygdala (Kleinhans et al. 2009). However, it is important to note that this study did not measure RS and did not compare the response to repeated presentations of the same face relative to presentation of different faces. Instead, they measured the change in amygdala response between the first and second run of the same scanning session. In this sense, their approach is comparable to studies measuring amygdala habituation to facial expressions (Breiter et al. 1996; Phillips et al. 2001), rather than RS studies. In addition, this study found no group difference in habituation to faces in the fusiform gyrus.

It is interesting to consider what might give rise to reduced RS effects. ASC has been associated with a bias towards local or feature-based processing, as opposed to global/holistic processing that characterizes neurotypical individuals (Happé and Frith 2006). Face perception depends more on holistic or configural processing than part-based processing (Maurer et al. 2002). Similarly, scenes are also composed of multiple component parts, and there is evidence that their overall meaning or “gist” is extracted before their component parts (Biederman 1981). Diminished RS to these categories in high autistic trait participants might therefore reflect a greater reliance on local features, or attention to different local features on each repetition of the same image. However, the observation that a negative relationship between autistic traits and RS also extends to simple geometric shapes, comprising a single contour and minimal local features, suggests that this is unlikely to be the case. It also seems unlikely this pattern can be accounted for by differences in attention. First, we found no relationship between AQ and RTs in the target detection task, whereas a positive relationship may be expected if high AQ participants were not attending to the stimulus. Second, if reduced RS is due to reduced attention then we should see the same pattern in all category-selective areas (i.e., all face-selective areas should be modulated by attention). Instead, reduced RS to faces was limited to specific regions (e.g., right FFA), rather than extending to the whole face-processing network. Finally, the pattern cannot be attributed to differential attention to the eye region, because an ANCOVA including eye tracking data indicated that dwell time on the eyes did not co-vary with RS.

RS has been posited to reflect various mechanisms. A common interpretation in neuroimaging is that it reflects fatigue of a neuronal population responding to a particular stimulus (Grill-Spector et al. 2006). One possibility, therefore, is that diminished RS in high autistic trait participants reflects differences in neuronal fatigue. A second proposal is that ASC is characterized by an atypical learning style in which each trained stimulus or stimulus feature is coded by separate, narrow, and nonoverlapping tuning functions (Qian and Lipkin 2011). While this allows relatively precise representations of each training example, it does not enable generalization to novel stimuli. This learning style contrasts with the typical learning style that deploys wider overlapping channels coding statistical regularities in the data that are optimized to generalize from training data to novel examples drawn from the same distribution. Qian and Lipkin (2011) propose that the ASC and typical learning styles favor low and high-dimensional feature spaces, respectively. In the ASC learning style, a low-dimensional feature space involves narrowly tuned channels with relatively few contributing cells. In contrast, a high-dimensional feature space involving broadly tuned channels has a greater number of contributing cells, and therefore a greater number of cells showing RS. Thus, narrow face tuning may also contribute to the reduced face aftereffects found in ASC (Pellicano et al. 2007), but the extent to which narrow tuning is associated with ASC remains to be determined.

A third alternative comes from predictive-coding theorists, who propose that RS is a consequence of a decrease in prediction error, that is, difference between bottom-up (stimulus-based) and top-down (prediction-based) inputs (Henson 2003; Friston 2005). Evidence in support of this position comes from several studies investigating RS in occipitotemporal cortex (Summerfield et al. 2008; Ewbank et al. 2011, 2013). Thus, diminished RS as a function of autistic traits could reflect differences in the intrinsic predictive-coding mechanisms of low and high autistic trait participants. This hypothesis accords with the proposal that some aspects of autism may reflect problems in utilizing prior knowledge (Mitchell and Ropar 2004; Gomot and Wicker 2012), which can be formalized as attenuated priors in a Bayesian framework (Pellicano and Burr 2012).

Across Experiments 1 and 2, the relationship between autistic traits and RS was more evident in the right than left FFA or right/left OFA. Experiment 2 showed that in addition to the FFA, a significant relationship between autistic traits and RS to faces was also found in bilateral OFA, a region thought to represent an earlier stage in the face-processing network. The relationship in OFA was not significant, however, when measuring RS using the AI. One interpretation of this finding is that compared with OFA, FFA may be more involved in the integration of face parts and the formation of an holistic face representation (Kanwisher and Yovel 2006); processes that may be compromised in individuals with greater numbers of autistic traits. For shapes, the relationship was more marked in right than left hemisphere regions. It is unclear why the relationship between AQ and RS is stronger in some ROIs than others, and additional work will be needed to explore this further. However, it is important to note that variation in the number of regions localized (i.e., each ROI was not localized in every participant) means that the number of participants included in the analyses varies for each ROI. Thus, differences in the spread of AQ scores and statistical power may partially account for regional differences in the strength of the relationship between AQ and RS.

Although it is unclear whether RS and perceptual aftereffects are a consequence of the same underlying mechanism, RS in category-selective cortical areas has been shown to be related to perceptual aftereffects resulting from adaptation to stimuli processed by those areas (Cziraki et al. 2010; Kaiser et al. 2013). Therefore, our finding of reduced RS in individuals with greater numbers of autistic traits for nonsocial (simple shapes and scenes) as well as social stimuli, suggests that aftereffects for both social and nonsocial stimuli may be reduced in such individuals. This hypothesis remains to be tested. However, a recent study found that children with ASC, who show smaller face distortion aftereffects than typical children, do not show reduced aftereffects for nonsocial stimuli, such as cars (Ewing et al. 2013a)). Whether aftereffects in ASC would nevertheless be reduced for other nonsocial stimuli (simple shapes and scenes) as used here remains to be seen. In addition, it is unclear whether the reduced face aftereffects found in children with ASC are also found in adults with ASC (Cook et al. 2014). Finally, it is also possible that there is a dissociation between RS and perceptual aftereffects, perhaps due to the different time scales (shorter for RS than for aftereffects) over which they are typically measured. Future studies will be needed to determine the precise relationship between neural RS and adaptation-induced perceptual aftereffects.

Finally, RS is not only found using block designs, but also occurs after a single repetition of a stimulus, with either no or multiple intervening stimuli (Grill-Spector et al. 2006). It would therefore be interesting to determine whether a relationship between autistic traits and RS is also observed using an event-related design involving single repetitions, where participants have fewer expectations about the nature of the next stimulus. Future studies will therefore be needed to elucidate the mechanisms that result in diminished RS in high autistic trait participants. Irrespective of the mechanisms involved; however, the present study highlights a core aspect of neural function that may be altered in individuals displaying increased autistic traits —namely, the way the brain responds to repeating/changing stimuli.

In conclusion, we have shown that individual differences in autistic traits predict RS to social and nonsocial visual objects, including both complex scenes and simple shapes, in category-selective areas. The results suggest that autistic traits are associated with discernible differences in the way the brain responds to repetition. Given the ubiquitous nature of RS in the brain, the finding that RS is diminished, or even abolished, in those with high autistic traits raises the possibility that differences in the basic neural mechanisms supporting RS might underpin core behavioral traits associated with autism, such as rigid and repetitive patterns of behavior and an insistence on sameness.

## Funding

This work was supported by the UK Medical Research Council under project code MC-A060-5PQ50 (A.J.C.), the Australian Research Council Centre of Excellence in Cognition and its Disorders (CE110001021), an ARC Professorial Fellowship to Rhodes (DP0877379) and an ARC Discovery Outstanding Researcher Award to Rhodes (DP130102300). S.B.C. was supported by the MRC, the Wellcome Trust, and the Autism Research Trust during the period of this work. Funding to pay the Open Access publication charges for this article was provided by the UK Medical Research Council under project code MC-A060-5PQ50.
